# High-sensitivity cardiac Troponin T delta concentration after repeat pulmonary vein isolation

**DOI:** 10.11613/BM.2019.020902

**Published:** 2019-06-15

**Authors:** Ivan Zeljkovic, Sven Knecht, Florian Spies, Tobias Reichlin, Beat Schaer, Stefan Osswald, Michael Kühne, Christian Sticherling

**Affiliations:** 1 Cardiology Department, University Hospital Basel, University of Basel, Basel, Switzerland; 2 Cardiovascular Research Institute Basel, Basel, Switzerland

**Keywords:** atrial fibrillation, pulmonary vein isolation, high-sensitivity troponin T, repeat procedure, recurrence

## Abstract

**Introduction:**

Difference between high-sensitivity cardiac troponin T concentrations (hs-cTnT) before and after ablation procedure (delta concentration) reflects the amount of myocardial injury. The aim of the study was to investigate hs-cTnT prognostic power for predicting atrial fibrillation (AF) recurrence after repeat pulmonary vein isolation (PVI) procedure.

**Materials and methods:**

Consecutive patients with paroxysmal AF undergoing repeat PVI using a focal radiofrequency catheter were included in the study. Hs-cTnT was measured before and 18-24 hours after the procedure. Standardized 3, 6 and 12-month follow-up was performed. Cox-regression analysis was used to identify predictors of AF recurrence.

**Results:**

A total of 105 patients undergoing repeat PVI were analysed (24% female, median age 61 years). Median (interquartile range) hs-cTnT delta after repeat PVI was 283 (127 - 489) ng/L. After a median follow-up of 12 months, AF recurred in 24 (23%) patients. A weak linear relationship between the total radiofrequency energy delivery time and delta hs-cTnT was observed (Pearson R^2^ = 0.31, P = 0.030). Delta Hs-cTnT was not identified as a significant long-term predictor of AF recurrence after repeated PVI (P = 0.920).

**Conclusion:**

This was the first study evaluating the prognostic power of delta hs-cTnT in predicting AF recurrence after repeat PVI. Delta hs-cTnT does not predict AF recurrence after repeat PVI procedures. Systematic measurement of hs-cTnT after repeat PVI does not add information relevant to outcome.

## Introduction

Pulmonary vein isolation (PVI) catheter ablation is an established therapeutic option for patients with atrial fibrillation (AF) (*1*). Various types of technologies for PVI are currently available, with focal radiofrequency (RF) and cryoballoon (CB) being the most frequently used (*1*, *2*). These two modalities have comparable rates of AF recurrence (*1*-*3*). The objective of current technological development is to decrease the AF recurrence rate by improving the durability of ablation lesions. Since cardiac troponin concentration measured before compared to troponin measured after ablation (delta concentration) is a reliable marker of myocardial injury, it has been studied as a long-term predictor of PVI success using different ablation modalities (*4*-*6*). The value of cardiac troponin T delta concentration as a long-term predictor after the index PVI was confirmed in several studies (*4*-*8*). The aim of the current study was to determine the prognostic power of high-sensitivity cardiac troponin T concentrations (hs-cTnT) in predicting AF recurrence after repeat PVI procedure during a long-term follow-up period.

## Materials and methods

### Study design and subjects

We conducted a single-centre, non-randomized, registry-based cohort study. Patients enrolled in the prospective Basel Atrial Fibrillation Pulmonary Vein Isolation (BEAT-AF-PVI) registry, in the period between January 2010 and March 2017, were eligible. Consecutive patients with paroxysmal AF, in whom the index PVI was performed using a focal RF or 2^nd^-generation CB and who underwent a repeat PVI procedure with focal RF regardless of the number of consecutive PVI procedures afterwards, were analysed. Indication for repeat procedure was recurrence of AF (at least three months after the last procedure which is defined as blanking period), but influenced by patient’s clinical symptoms and physicians discretion. Patients with non-paroxysmal type of AF, patients in whom additional linear lesions in left atrium were done at any procedure, in whom the repeat PVI procedure was not done using focal RF ablation with three-dimensional (3D) mapping system, and those who did not sign informed consent for participating in the registry were excluded from the study.

Baseline demographic characteristics, medical history and medication were collected. Transthoracic and transoesophageal echocardiogram to rule out left atrial (LA) thrombus, to determine LA diameter in the parasternal long axis (PLAX) plane and left ventricular ejection fraction (LVEF) were performed before the repeat PVI.

All included patients gave written informed consent for participating in the BEAT-AF-PVI registry. The local Ethics Committee gave its approval for the study, which was conducted in accordance with the current version of Declaration of Helsinki.

### Ablation procedure

Index PVI was done using focal RF ablation catheter (Navistar Thermocool or Navistar Thermocool SmartTouch, Biosense Webster, Diamond Bar, USA) in combination with a 3D mapping system (Carto3, Biosense Webster, Diamond Bar, USA) or using 2^nd^-generation CB catheter (ArcticFront Advance 23 mm or 28 mm, Medtronic Inc., Minneapolis, USA) as described in detail earlier (*1*, *2*, *6*). The same focal irrigated-tip RF catheter in combination with a 3D mapping system were used to perform repeat PVI (*2*, *6*). Radiofrequency energy was delivered with 25 Watts (W) at the posterior wall and 25W or 30W at the anterior wall for a duration 20-40 seconds.

An electrical reconnection site was defined as the location in the previous ablation line that was associated with the site of earliest activation as assessed by the circular mapping catheter, which was placed just distal from the previous (existing) ablation line. Reconnected vein was defined as a pulmonary vein (PV) with one or more electrical reconnection sites after the initial ablation during which a circular continuous line around left and right pulmonary veins was made and no permanent scar was achieved at that site. During repeat PVI, all PVs were assessed for the presence of electrical reconnection site/s and reconnection gaps were assessed based on the signals on a circular diagnostic catheter (Lasso Nav, Biosense Webster, Diamond Bar, USA) placed at the ostium of individual PV. In addition, the operator determined the reconnection gap, based on the activation sequence and the amount of ablation needed to achieve PV re-isolation. The reconnection gap was defined as focal if only one or two focal ablations were performed or as segmental if ≥ 3 focal ablations were performed to achieve complete PV re-isolation. The repeat PVI end-point was the documentation of PV bidirectional block using the circular diagnostic catheter (Lasso Nav, Biosense Webster, Diamond Bar, USA).

### Outcome and follow-up

Primary end-point was the AF recurrence during the entire follow-up period. Episodes of AF or left atrial tachycardia lasting > 30 seconds were considered as an AF recurrence. Follow-up was performed at 3, 6 and 12 months after repeat PVI procedure with 12-lead ECG, 24-hour and 7-day Holter-electrocardiogram (ECG) monitoring, and afterwards yearly with telephone interviews and collecting data from patients’ cardiologists.

### hs-cTnT analysis

Blood samples were collected in the morning before the procedure and 18-24 hours after the procedure using lithium-heparin-gel blood collection tubes (S-Monovette, Sarstedt AG&Co., Nümbrecht, Germany). The hs-cTnT concentrations were determined with modified fourth-generation assay on Cobas e411 System (Roche Diagnostics GmbH, Mannheim, Germany) and Modular Analytics E170/cobas e 601 immunoanalysers (Roche Diagnostics GmbH, Mannheim, Germany) according to the instructions of the manufacturer. Detection is based on an electrochemiluminescence immunoassay (ECLIA) (*9*, *10*). The laboratory sets the normal value at a 99^th^ percentile concentration of 14 ng/L and a corresponding coefficient of variation (CV) of 10% at 13 ng/L (*9*). The limit of blank (LoB) and limit of detection (LoD) have been determined to be 3 ng/L and 5 ng/L, in accordance with Clinical and Laboratory Standards Institute (CLSI) EP17-A requirements (*9*). Lyophilized human serum matrix with added human recombinant troponin T (PreciControl Troponin, Roche Diagnostics GmbH, Mannheim, Germany) in two concentration ranges (28.5 ng/L and 2510 ng/L) was used as internal quality control material during the study. Between-run imprecision for the hs-cTnT assay during the study period was 8.4% at 28.5 ng/L and 4.9% at 2510 ng/L. From the imprecision profile the 10% CV corresponded to a cTnT concentration of 11.9 ng/L which was below the 99th percentile of the reference population. This study did not include measurements with hs-cTnT lots that required the revision of the calibration curve (*10*). Ablation related hs-cTnT delta concentration was defined as the difference between the two hs-cTnT measurements.

### Statistical analysis

The distribution of variables was tested using Kolmogorov-Smirnov test. Continuous variables are presented as mean ± standard deviation when variable’s distribution was normal and as median with interquartile range (IQR) when variable’s distribution was skewed. For continuous variables, comparisons were made using Student’s T-test as parametric test for independent samples, or using Mann-Whitney U test as non-parametric test for independent samples. Categorical variables are presented as absolute numbers and/or percentages. Discrete variables were compared using Fisher’s exact test. Association between hs-cTnT delta concentration and other parameters were tested using Spearman’s correlation and linear regression analysis without intercept. Cox regression models using a stepwise forward procedure were constructed to assess the associations between the hs-cTnT delta concentration and the repeat PVI outcome. A P-value of < 0.05 was predefined to indicate statistical significance. Statistical analysis was performed using SPSS version 20 (IBM SPSS Statistics, Armonk, New York, USA).

## Results

### Baseline data

One hundred five Caucasian patients with recurrence of paroxysmal AF and repeat PVI were included in the study. Median age of the study population was 61 years (range 33 - 78), 24% were female. Median follow-up was 362 (IQR 160 - 446) days. For the index PVI, focal RF or CB ablation were used in 81 and 24 patients, respectively. Baseline characteristics of the study group before the repeat PVI are given in Table 1.

**Table 1:** Baseline characteristics of the study group before repeat PVI

**Study group (N = 105)**	
**Demographics**	
Age, years	61 (33 - 78)
Males, N (%)	80 (76)
BMI, kg/m^2^	27 ± 4
**History**	
Hypertension, N (%)	54 (51)
Diabetes mellitus, N (%)	9 (8.6)
Hyperlipidaemia, N (%)	24 (23)
Smoking, N (%)	10 (9.5)
Cerebral stroke, N (%)	7 (6.7)
Coronary artery disease, N (%)	7 (6.7)
Myocardial infarction, N (%)	2 (1.9)
Atrial flutter, N (%)	21 (20)
AF history, months	36 (3 - 106)
HAS BLED score	0.9 ± 0.9
CHA_2_DS_2_VASc score	1.6 ± 1.5
**Echocardiography before repeat PVI**	
LA diameter (in PLAX), mm	39 ± 7
LVEF, %	58 ± 8
LA volume index, mL/m^2^	35 ± 11
**Laboratory results**	
Haemoglobin, g/L	148 (142 - 157)
Creatinine, µmol/L	80 (71 - 93)
hs-cTnT before PVI, ng/L	7 (5 - 10)
hs-cTnT after PVI, ng/L	291 (136 - 496)
C-reactive protein, mg/L	1.1 (0.5 - 2.7)
Age is presented as median (range). Continuous variables are represented as mean ± standard deviation or median (interquartile range). BMI – body mass index. AF – atrial fibrillation. PVI – pulmonary vein isolation. LA – left atrium. PLAX – parasternal long axis. LVEF – left ventricular ejection fraction. hs-cTnT – high sensitive cardiac Troponin T. HAS BLED score is calculated as follows: hypertension (uncontrolled > 160 mmHg systolic arterial blood pressure) = 1 point, abnormal renal and/or liver function = 1 point + 1 point, prior history of stroke = 1 point, prior major bleeding or predisposition to bleeding = 1 point, labile INR (Unstable/high INR and/or Time in Therapeutic Range < 60%) = 1 point, age > 65 years = 1 point, prior alcohol/drug usage history (≥ 8 drinks/week) and/or medication usage predisposing to bleeding (antiplatelet agents, NSAIDs) = 1 point + 1 point. HAS BLED score has a minimum of 0 to maximum 9 points. CHA_2_DS_2_VASc score is calculated as follows: congestive heart failure (or left ventricular systolic dysfunction) = 1 point, hypertension (blood pressure consistently above 140/90 mmHg or treated hypertension on medication) = 1 point, age 65–74 years = 1 point or age ≥ 75 years = 2 points, diabetes mellitus = 1, prior stroke or transient ischemic attack or thromboembolism = 2 points, vascular disease (*e.g.* peripheral artery disease, myocardial infarction, aortic plaque) = 1 point, female = 1 point. CHA_2_DS_2_VASc score has a minimum 0 point to maximum 9 points.	

### hs-cTnT analysis

High-sensitivity cardiac troponin T concentrations were higher in all patients after repeat PVI. Median hs-cTnT concentration before repeat PVI was 7 (5 - 10) ng/L and after the procedure 291 (125 - 490) ng/L, with a delta hs-cTnT of 283 (127 - 489) ng/L. A linear relationship between the RF delivery time and the delta hs-cTnT was observed for the entire dataset (Pearson R^2^ = 0.31, P = 0.030). Selective analysis showed a weak correlation for patients that underwent index PVI using focal RF (Pearson R^2^ = 0.29, P = 0.014) and no significant correlation for patients that underwent index PVI using 2^nd^-generation CB ablation (Pearson R^2^ = 0.42, P = 0.080). In addition, there was no influence of cardiovascular risk factors on delta hs-cTnT, including diabetes mellitus (300 (189 - 547) ng/L in diabetic patients *vs.* 279 (127 - 470) ng/L in non-diabetic patients, P = 0.330).

Only one reconnected PV was assessed during repeat PVI in 21 patient, and two, three, and four reconnected PVs in 41, 22 and 14 patients, respectively. Seven patients had all PVs isolated. There was no significant correlation between the number of reconnected PVs and delta hs-cTnT after repeat PVI (P = 0.810). Among patients with PV reconnection, 30 patients (31%) had focal type of reconnection gaps and 68 had segmental gaps, respectively. There was no difference between the patients with focal and segmental reconnection gaps regarding the delta hs-cTnT after procedure (focal 270 (96 – 403) ng/L *vs.* segmental 295 (144 - 518) ng/L, P = 0.370).

### Delta hs-cTnT and AF recurrence

Acute procedural success at repeat PVI procedure was achieved in all study patients. Ninety-eight patients (93%) had at least 6-month follow-up. During the median follow-up period of 362 (160 - 446) days after the repeat procedure, AF recurrence was observed in 24 patients (23%). Delta hs-cTnT with the repeat PVI was not different between the patients with and without AF recurrence (284 (115 - 671) *vs.* 285 (132 - 456) ng/L, P = 0.780). In addition, there was no significant difference in delta hs-cTnT between patients that underwent index PVI using focal RF and 2^nd^-generation CB ablation (296 (167 - 520) *vs.* 267 (167 - 426) ng/L, P = 0.070). Multivariable Cox regression analysis (corrected for sex, age, body mass index) did not reveal delta hs-cTnT as a significant predictor of AF recurrence after repeat PVI (odds ratio (OR) 1.00, 95% confidence interval (CI) 1.00 - 1.00, P = 0.750). In addition, cumulative delta hs-cTnT (sum of delta hs-cTnT after index and repeat PVI procedures) did not show significant correlation with AF recurrence after repeat PVI (OR 1.08, 95% CI 0.94 - 1.14, P = 0.680).

## Discussion

This single-centre, non-randomized registry-based study investigated the prognostic power of delta hs-cTnT to predict AF recurrence after repeat PVI in patients with paroxysmal AF. To our knowledge, this is the first study assessing prognostic power of delta hs-cTnT after repeat PVI. The main findings of this study are: 1) delta hs-cTnT after repeat PVI was not identified as a long-term predictor of AF recurrence; 2) a weak correlation between the delta hs-cTnT and RF delivery time at the repeat PVI was determined.

In this study, a substantial delta hs-cTnT with the repeat PVI ablation was observed. We confirmed the relationship between total RF delivery time and delta hs-cTnT, as reported previously for index PVI procedures (*6*, *11*). However, this correlation was weak, and after subanalysis no significant relationship was found in patients that underwent index PVI using CB ablation. This could be explained by the fact that during the repeat PVI procedure most of the ablation is performed at already ablated area of the PV ostia where there is probably less myocardial and more fibrotic tissue as well as that the total RF time is lower in comparison to index PVI. In addition, the number of reconnected PVs did not influence delta hs-cTnT, which speaks in favour of the above mentioned hypothesis.

Various biomarkers (cardiac troponin I, creatine kinase, creatine kinase - isoenzyme MB (muscle/brain), high-sensitive cardiac troponin T, high-sensitive C-reactive protein, plasma B-type natriuretic peptide, *etc.*) were studied as long-term predictors of AF recurrence after the index PVI (*4*-*8*). Information about delta hs-cTnT after repeat PVI was missing. Despite the proven capability of delta hs-cTnT to quantify myocardial injury after catheter ablation, controversial results have been reported to predict AF recurrence after PVI procedure (*4*-*8*, *11*). This is probably because LA myocardial injury is a very unspecific marker for the efficacy or durability of the PV isolation. Moreover, delta hs-cTnT was not identified as a long-term predictor of repeat PVI efficacy in the present study. In addition, not even the type of the reconnection gap was correlated to the delta hs-cTnT, which could point to the fact that we ablate more than necessary to achieve PV re-isolation with additional antral ablation after achieving re-isolation. Reasons in favour of the aforementioned fact could be that delta hs-cTnT does not reflect continuity of the PVI lesions, but only the amount of destroyed myocardial cells. Ultimately, AF recurrence might be due to non-PV triggers, which is not addressed by PV isolation. In the end, our findings raise the question of the clinical value of hs-cTnT measurement in periprocedural management of patients undergoing PVI, especially repeat procedures. It is well known that following PVI ablation hs-cTnT will increase, but the amount of the delta concentration does not give us any additional useful information (*5*, *12*).

The results of the present study should be interpreted in the light of several limitations. Firstly, this was a single-centre experience performed on a relatively small group of patients, which limits the strength of the observations. Secondly, gap identification and classification was performed based on the circular diagnostic catheter potentials and activation sequence. However, activation mapping using novel electro-mapping systems could have yielded results that are more precise to identify the gap in the circumferential lesion around the PV. Thirdly, we did not use implantable loop-recorders for AF recurrence assessing, consequently probably missing some asymptomatic AF episodes that could affect the reported AF recurrence rate. Finally, the procedural data analysis of the third PVI procedure was not performed determining the value of delta hs-cTnT as predictor of PV reconnection after the repeat PVI.

In conclusion, to our knowledge, this is the first study assessing delta hs-cTnT after the repeat PVI. Delta hs-cTnT was not identified as a long-term predictor of AF recurrence after repeat PVI. Systematic measurement of hs-cTnT concentration after repeat PVI does not add additional information relevant to long-term outcome.
